# Efficacy of intracellular immune checkpoint-silenced DC vaccine

**DOI:** 10.1172/jci.insight.98368

**Published:** 2018-02-08

**Authors:** Danhong Wang, Xue F. Huang, Bangxing Hong, Xiao-Tong Song, Liangding Hu, Min Jiang, Bin Zhang, Hongmei Ning, Yuhang Li, Chen Xu, Xiao Lou, Botao Li, Zhiyong Yu, Jiangwei Hu, Jianlin Chen, Fan Yang, Haiyan Gao, Guoliang Ding, Lianming Liao, Lisa Rollins, Lindsey Jones, Si-Yi Chen, Hu Chen

**Affiliations:** 1 Department of Hematopoietic Stem Cell Transplantation, Affiliated Hospital of Academy of Military Medical Sciences, Beijing, China.; 2 Department of Molecular Microbiology and Immunology and Norris Comprehensive Cancer Center, University of Southern California, Los Angeles, California, USA.; 3 Center for Cell and Gene Therapy, Baylor College of Medicine, Houston, Texas, USA.; 4 Department of Oncology, Academy of Integrative Medicine, Fujian University of Traditional Chinese Medicine, Fuzhou, China.

**Keywords:** Hematology, Immunology, Dendritic cells, Immunotherapy, Leukemias

## Abstract

**BACKGROUND.** DC-based tumor vaccines have had limited clinical success thus far. SOCS1, a key inhibitor of inflammatory cytokine signaling, is an immune checkpoint regulator that limits DC immunopotency.

**METHODS.** We generated a genetically modified DC (gmDC) vaccine to perform immunotherapy. The adenovirus (Ad-siSSF) delivers two tumor-associated antigens (TAAs), survivin and MUC1; secretory bacterial flagellin for DC maturation; and an RNA interference moiety to suppress SOCS1. A 2-stage phase I trial was performed for patients with relapsed acute leukemia after allogenic hematopoietic stem cell transplantation: in stage 1, we compared the safety and efficacy between gmDC treatment (23 patients) and standard donor lymphocyte infusion (25 patients); in stage 2, we tested the efficacy of the gmDC vaccine for 12 acute myeloid leukemia (AML) patients with early molecular relapse.

**RESULTS.** gmDCs elicited potent TAA-specific CTL responses in vitro, and the immunostimulatory activity of gmDC vaccination was demonstrated in rhesus monkeys. A stage 1 study established that this combinatory gmDC vaccine is safe in acute leukemia patients and yielded improved survival rate. In stage 2, we observed a complete remission rate of 83% in 12 relapsed AML patients. Overall, no grade 3 or grade 4 graft-versus-host disease incidence was detected in any of the 35 patients enrolled.

**CONCLUSIONS.** This study, with combinatory modifications in DCs, demonstrates the safety and efficacy of SOCS1-silenced DCs in treating relapsed acute leukemia.

**TRIAL REGISTRATION.** ClinicalTrials.gov NCT01956630.

**FUNDING.** National Institute of Health (R01CA90427); the Key New Drug Development and Manufacturing Program of the “Twelfth Five-Year Plan” of China (2011ZX09102-001-29); and Clinical Application Research of Beijing (Z131107002213148).

## Abstract 2

A phase I trial demonstrates the safety and efficacy of SOCS1-silenced DCs in treating relapsed acute leukemia.

## Introduction

Acute leukemia (AL) is characterized by a differentiation block in early hematopoietic progenitors and a rapid increase in blast cells, which leads to an accumulation of immature cells in the bone marrow and peripheral blood. AL treatment outcomes have not improved in the past 2 decades, as evidenced by high relapse rates (1, 2). Relapse is caused by the persistence of malignant cells, designated as minimal residual disease, after complete remission (CR). Despite the high morbidity and mortality rates, allogeneic hematopoietic stem cell transplantation (allo-HSCT) is the first-line treatment for AL due to relatively lower relapse and higher survival rates associated with the graft-versus-leukemia effect exerted by donor T lymphocytes (3, 4). Treatment for relapsed AL includes a second allo-HSCT and donor lymphocyte infusion (DLI). While the overall remission and 2-year overall survival (OS) rates for DLI are between 15%–42% and 15–20%, respectively, the incidence of DLI-related graft-versus-host disease (GVHD) is high (40%–60%) (5, 6).

Recently, several immunotherapeutic strategies have been developed to improve antileukemic immunity against AL, including the use of DCs. DCs are the most potent professional antigen-presenting cells, and they have a unique capacity to prime adaptive immune responses against microbial pathogens and tumor-associated antigens (TAAs) (7, 8). As such, DC-based vaccines have been extensively tested in mouse tumor models and in clinical trials (8–11). DC vaccines can activate TAA-specific effector T cells that can effectively target and kill tumor cells, induce immunological memory to prevent tumor relapse (12), and have generally exhibited good safety profiles in phase I clinical trials. For example, a clinical trial with prostate cancer patients demonstrated increased survival after vaccination with ex vivo TAA-pulsed DCs (13). Despite these promising results, DC vaccines rarely cause tumor regression (an objective clinical response) (11), suggesting that self-tolerance at the host level may be maintained in vaccinated patients. Additional explanations for the limited effectiveness of DC vaccines include incomplete DC activation, inefficient antigen loading, the heterogeneous nature of DC populations, tumor-mediated immunosuppression, self-tolerance against TAAs, low-avidity T cells to TAAs, and suppression by Tregs. More recently, genetic studies in mice have demonstrated that negative regulators of proinflammatory signal transduction pathways, known commonly as immune checkpoints, are essential for the maintenance of self-tolerance and are a limiting factor in immune responses (14, 15). Thus, understanding and disabling these immune checkpoints may be key to enhancing the clinical efficacy of DC-based tumor vaccines.

Previous studies from our laboratory have revealed a critical role for SOCS1 as an intracellular immune checkpoint molecule that contributes to maintaining self-tolerance and limiting antitumor immunity (16, 17). SOCS1 is an essential negative regulator of proinflammatory signaling in T cells and antigen-presenting cells and negatively regulates TLR signaling (18–20). We previously demonstrated that SOCS1 silencing via shRNA enhanced the immunopotency of DCs, leading to the breakage of self-tolerance at the host level and induction of antigen-specific CTL responses capable of controlling preestablished, weakly immunogenic tumors in mice (16, 17, 21). However, inhibition of SOCS1 negative feedback alone is insufficient to activate DCs, since proinflammatory stimuli, such as TLR agonists, are required to initiate signaling cascades that lead to DC maturation. After ligand binding, TLR signaling activates transcription factors, which then induce the expression of a large number of proinflammatory genes encoding various cytokines, chemokines, and costimulatory molecules. In prior mouse tumor model studies, we demonstrated that SOCS1 shRNA-silenced DCs stimulated with TLR agonists, including LPS and polyinosinic/polycytidylic acid, were able to persistently induce CTLs, which resulted in effective antitumor responses (16, 17).

The filamentous protein flagellin of bacterial flagella, a TLR5 agonist (22), induces the maturation of and chemokine production by human DCs (hDCs) and directly activates NK and T cells (23–26). Recombinant flagellin-antigen fusion proteins have been shown to enhance the immunogenicity of coexpressed antigens and induce potent T cell and antibody responses in mice and monkeys (27–33). Moreover, DNA vectors coexpressing flagellin with antigens significantly enhanced antigen-specific T cell and antibody responses in mice (34). Although flagellin is a potent adjuvant for stimulating antigen-specific cellular and humoral immune responses, we and others have demonstrated that stimulation with TLR agonists alone is not enough to break self-tolerance at the host level or to induce effective antitumor activity (14, 17). Therefore, in the current study, we designed a DC vaccine that combines SOCS1 inhibition and flagellin immune stimulation.

Survivin, a member of the inhibitor of apoptosis family of proteins, and mucin 1 (MUC1), a cell-surface glycoprotein, are TAAs that are expressed by a broad range of human cancers that cause significant morbidity and mortality worldwide. For example, though undetectable in normal tissues, survivin was shown to be expressed by all 60 tumor cell lines used for drug screening by the National Cancer Institute (35) and in a vast majority of cancers in vivo (36), including melanoma (37), sarcoma (38), and leukemias (39). As such, survivin has been considered a universal anticancer target (40). MUC1 is also overexpressed in a number of human tumors (41). For example, high surface expression of MUC1 was shown on the cells of approximately 50% of AL patients (42). Thus, MUC1 does not require presentation by MHC molecules to be recognized for antibody-mediated tumor destruction. For these reasons, we designed our DC vaccine to target these two TAAs to allow for broad activity against various types of human cancer.

In the current study, we designed an adenoviral vector (Ad-siSSF) encoding SOCS1 shRNA as a checkpoint inhibitor, a survivin-MUC1 fusion protein as a source of TAAs, and bacterial flagellin as a TLR5 agonist for use in a genetically modified DC (gmDC) vaccine platform (referred to hereafter as Ad-siSSF-DC). We then characterized this vaccine, investigated its potential to induce CTL responses in vitro, and performed a preclinical safety study in rhesus monkeys. Subsequently, a 2-phase clinical trial was conducted. The first phase of the trial was performed in 48 patients with either acute lymphoblastic leukemia (ALL) or acute myeloid leukemia (AML) to compare the safety and efficacy between autologous Ad-siSSF-DC treatment and standard DLI by measuring OS and GVHD incidence in vivo. The second phase of the trial was performed in an additional 12 AML patients to evaluate the clinical efficacy of the Ad-siSSF-DC vaccine and any associated adverse events.

## Results

### Ad-siSSF efficiently activates hDCs in vitro.

**Figure 1:**
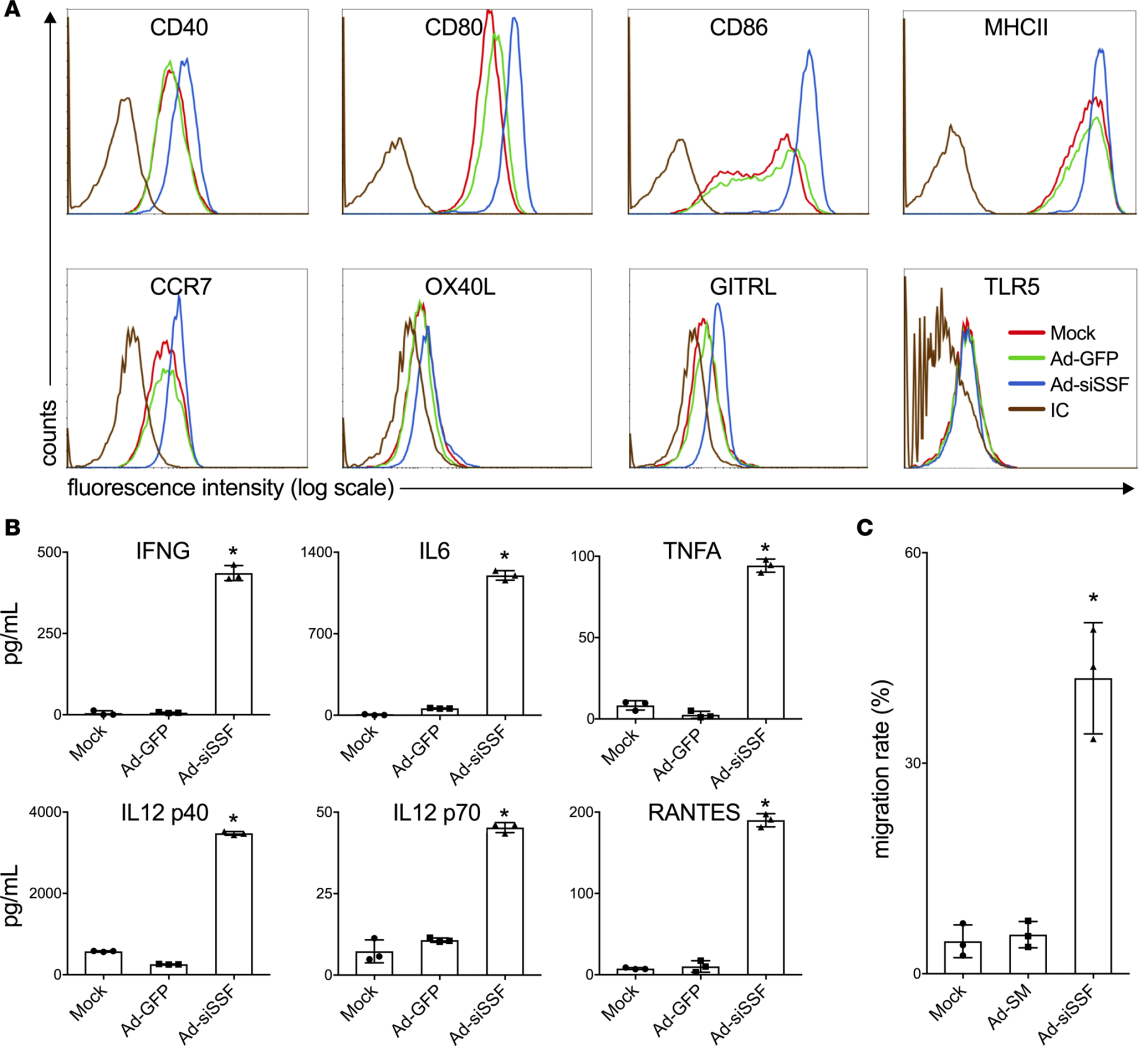
Enhanced maturation of Ad-siSSF–transduced hDCs. (**A**) Expression levels of a panel of surface markers on human PBMC DCs at 48 hours after transduction with Ad-siSSF, control Ad-GFP virus (MOI = 25,000 vp), or PBS-treated (mock) (isotype controls: brown line). With the exception of OX40L and TLR5, all surface markers are upregulated after Ad-siSSF transduction compared with mock or Ad-GFP controls. (**B**) The levels of representative proinflammatory cytokines and chemokines in the culture media of human PBMC DCs 48 hours after transduction with different adenoviruses at a MOI of 25,000 vp, as analyzed by ELISA. The hDCs have significantly enhanced levels of these cytokines and chemokines after Ad-siSSF transduction compared with controls. (**C**) Migration rates of transduced human PBMC DCs in response to recombinant CCL21 (100 ng/ml) show the enhanced migratory activity of Ad-siSSF–transduced DCs compared with controls. Results are presented from 1 of 3 repeated assays (mean ± SEM). *P < 0.05, Ad-siSSF vs. Ad-SM DCs, as determined by Student’s 2-tailed *t* test.

We generated a recombinant replication-deficient adenoviral vector, designated Ad-siSSF, which coexpresses a human SOCS1 shRNA (siSOCS1), dominant-negative survivin-MUC1 fusion protein (SM), and the TLR5 agonist secretory flagellin (Supplemental Figure 1A; supplemental material available online with this article; https://doi.org/10.1172/jci.insight.98368DS1). The ability of the Ad-siSSF virus to efficiently coexpress all three of these components was demonstrated via Western blot analysis (Supplemental Figure 1, B–D). We next investigated the effect of Ad-siSSF transduction on the maturation of peripheral blood mononuclear cell–derived (PBMC-derived) hDCs. Surface expression of multiple costimulatory molecules (CD40, CD80, and CD86) and MHC class II molecules was markedly increased on Ad-siSSF–transduced DCs compared with DCs transduced with a recombinant adenovirus encoding only the SM fusion protein (Ad-SM) or an adenovirus encoding GFP (Ad-GFP) (Figure 1A). Surface levels of glucocorticoid-induced tumor necrosis factor receptor (GITR) ligand and OX40 ligand, a T cell costimulatory signal ligand, were also obviously enhanced on Ad-siSSF-DCs (Figure 1A). Next, hDCs were transduced with Ad-siSSF or control adenoviruses, and culture media were collected at different time points after transfection for cytokine analysis by ELISA. This method revealed increased secretion of proinflammatory cytokines and chemokines, including IL12p40, IL12p70, IFNG, IL6, TNFA, and RANTES, by Ad-siSSF-DCs (Figure 1B). To confirm this, we further assessed the effect of Ad-siSSF transduction on cytokine production by hDCs using an array to detect 42 cytokines, and we again found that the secretion of various proinflammatory cytokines was increased in Ad-siSSF–transduced hDCs (Supplemental Figure 2). Taken together, these results indicate that Ad-siSSF transduction is an effective method to phenotypically activate PBMC-derived hDCs.

The migration of mature antigen-presenting DCs to lymph nodes for antigen-specific T and B cell priming is critical for the induction of antitumor immunity. After maturation, DCs express the lymph node–homing receptor CCR7, which enables cell migration toward its CCL19 and/or CCL21 ligands (43). We observed that CCR7 expression was substantially upregulated on Ad-siSSF–transduced DCs compared with that on control Ad-GFP-DCs (Figure 1A). Next, we examined the migratory ability of Ad-siSSF-DCs using a standard Transwell in vitro migration assay (44). This revealed a significant increase in the migration of Ad-siSSF–transduced DCs compared with that of Ad-SM–transduced DCs, suggesting the functional activation of Ad-siSSF–transduced DCs (Figure 1C).

### Ad-siSSF–transduced hDCs potently induce TAA-specific CTL responses in vitro.

**Figure 2:**
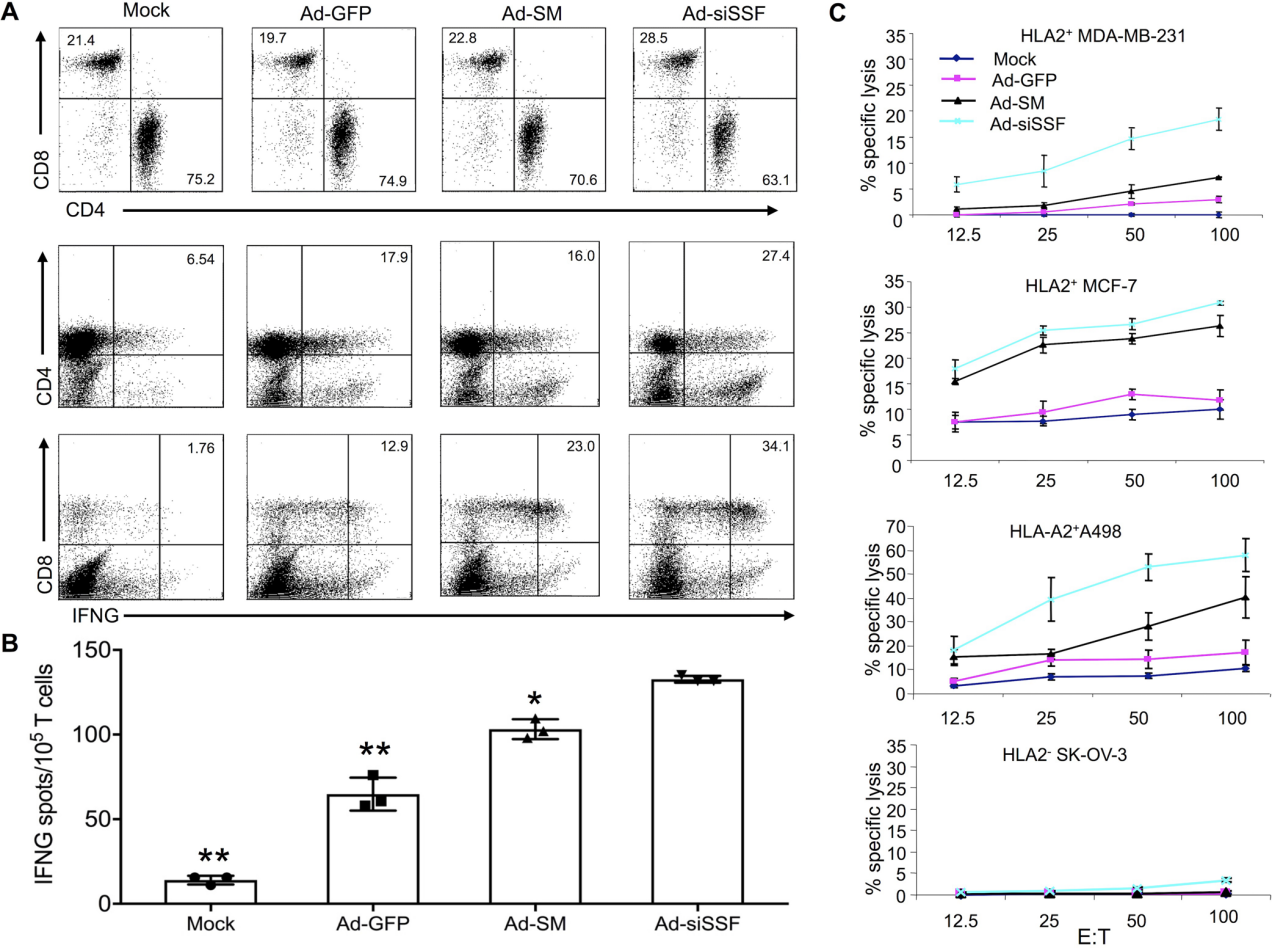
Enhanced priming of TAA-specific T cells by Ad-siSSF-DCs and enhanced TAA-specific CTL cytolytic activity against human tumor cells. Human autologous T cells were cocultured with Ad-transduced DCs or PBS-treated hDCs (mock) (20:1) for 2 weeks. (**A**) Intracellular IFNG staining of cocultured CD4^+^ and CD8^+^ T cells. FACS analysis shows an increase of IFNG expression after coculture with Ad-siSSF-DCs compared with controls. (**B**) Increased IFNG-producing T cells after coculture with Ad-siSSF-DCs compared with coculture with controls is shown by ELISpot. Representative data from 1 of 5 HLA-A2^+^ donors are shown. (**C**) Cytolytic activities of HLA-A2^+^ T cells against various human tumor cell lines after 2 weeks of in vitro sensitization with different adenovirus-transfected autologous DCs were determined by standard ^51^Cr release assays. T cells sensitized by Ad-siSSF-DCs exhibited increased killing compared with those sensitized by controls. Cytolytic percentages are presented from 1 of 3 repeated experiments. Error bars represent mean ± SEM. *P < 0.05, Ad-siSSF vs. Ad-SM; **P < 0.01, Ad-siSSF vs. Ad-GFP or mock, as determined by Student’s 2-tailed *t* test.

The enhanced maturation of Ad-siSSF-DCs prompted us to test their ability to prime TAA-specific CTLs using in vitro T cell immunization assays in which Ad-transduced HLA-A2^+^ PBMC-derived hDCs were cocultured with autologous T cells, and the numbers of TAA-specific T cells (i.e., against survivin or MUC1 epitopes) were determined by IFNG ELISpot and intracellular staining assays. These experiments showed increased percentages of IFN-producing CD8^+^ T cells and CD4^+^ T cells in cocultures with Ad-siSSF-DCs compared with Ad-SM-DC cocultures (Figure 2A). Consistent with this, IFNG ELISpot assays also revealed increased numbers of IFNG-producing T cells in the Ad-siSSF-DC cocultures compared with controls (Figure 2B). These in vitro results indicate that DCs transduced with Ad-siSSF have a strong ability to prime antigen-specific CTL responses. We further tested the cytolytic activity of the primed T cells against multiple HLA-A2^+^ tumor cells lines (breast MDA-MD-231 and MCF-7 and renal A-498) and an HLA-A2^-^ cell line (SK-OV-3), all of which are MUC1^+^ (Supplemental Figure 3), using a standard chromium release assay. Indeed, T cells primed with Ad-siSSF-DCs had more potent cytolytic activity against MCF-7, MDA-MB-231, and A-498 cells than those primed with Ad-SM-DCs (Figure 2C). The specificity of this cytotoxic response was demonstrated by the inability of the primed T cells to kill SK-OV-3 cells. These results demonstrate the powerful immunopotency of Ad-siSSF–transfected DCs to induce TAA-specific CTL responses capable of selectively killing human tumor cells.

### Ad-siSSF induces TAA-specific responses in monkeys.

**Figure 3:**
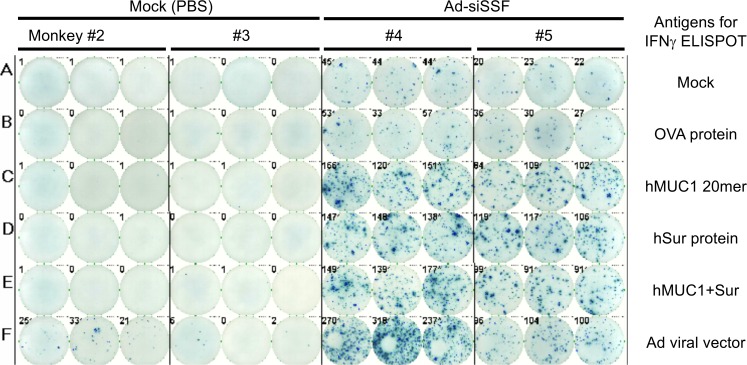
ELISpot assay of immune responses induced by a single injection of Ad-siSSF vector in monkeys. Groups of monkeys (*n* = 3 per group) were injected with Ad-siSSF or control PBS, and toxicities and immune responses were measured 14 days later. Frequencies of antigen-specific T cells against MUC1 (hMUC1 20mer), survivin (hSur protein), and against the MUC1-survivin fusion protein (hMUC1+Sur) were significantly increased in monkeys given Ad-siSSF compared with the mock group (PBS). *P* < 0.00001 for responses against designated peptides in the Ad-siSSF group vs. mock group, as determined by Student’s 2-tailed *t* test.

In order to preclinically assess the toxicity and immunopotency of the Ad-siSSF vaccine in vivo, two groups of rhesus monkeys (~3 years old, *n* = 3 per group) were intramuscularly injected once with the recombinant Ad-siSSF vector or PBS. Necropsy analyses on day 14 after injection revealed that there were no apparent toxicities associated with the Ad-siSSF vector. We then examined the potential immune responses induced by this single Ad-siSSF injection using an ELISpot assay. As shown in Figure 3, the frequencies of survivin- and MUC1-specific T cells were significantly increased in monkeys treated with Ad-siSSF compared with controls, indicating that Ad-siSSF is capable of activating TAA-specific T cells in primates.

### Autologous Ad-siSSF-DCs increase survival of patients with AL.

**Figure 4:**
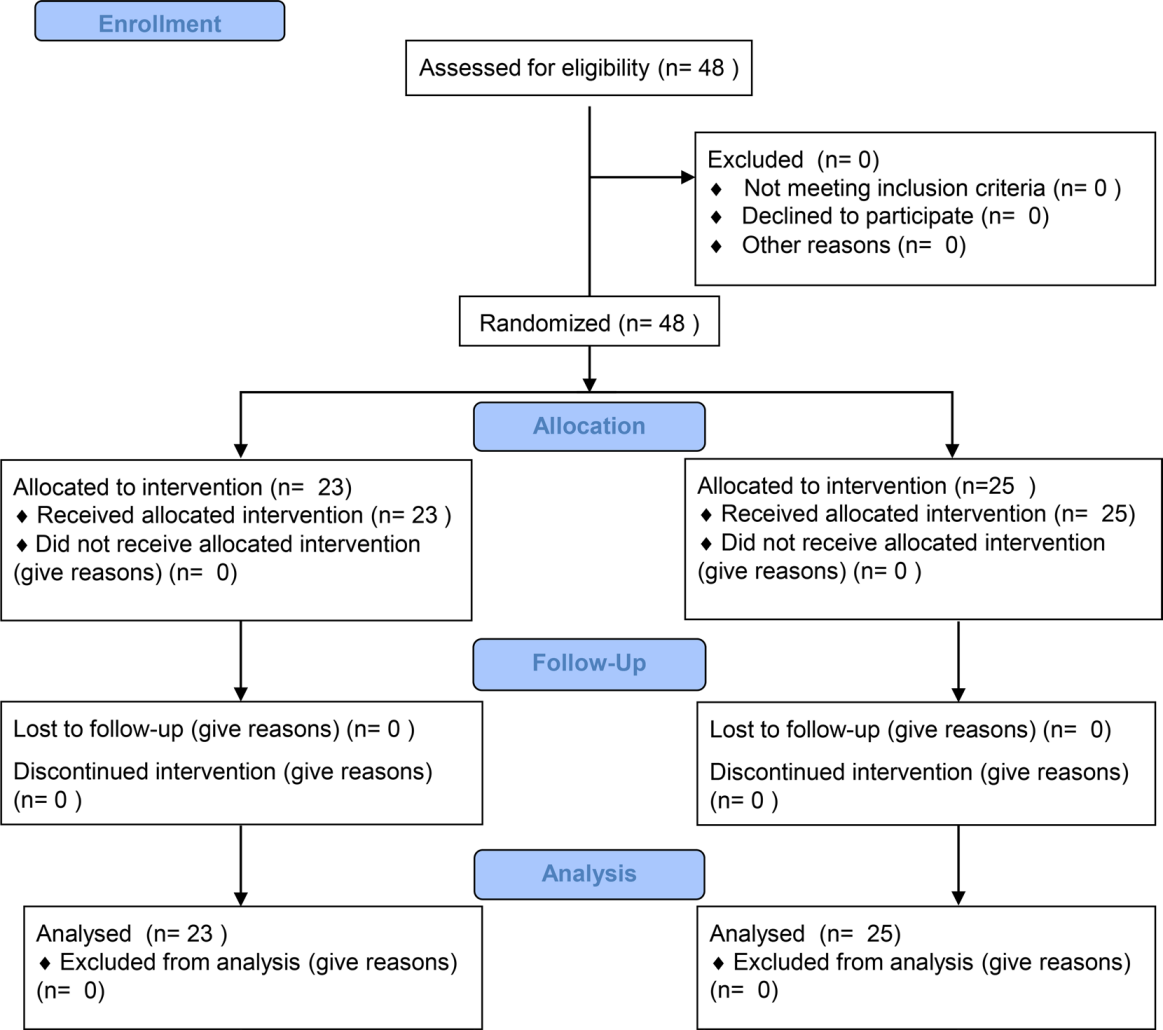
Consolidated Standards of Reporting Trials diagram.

**Figure 5:**
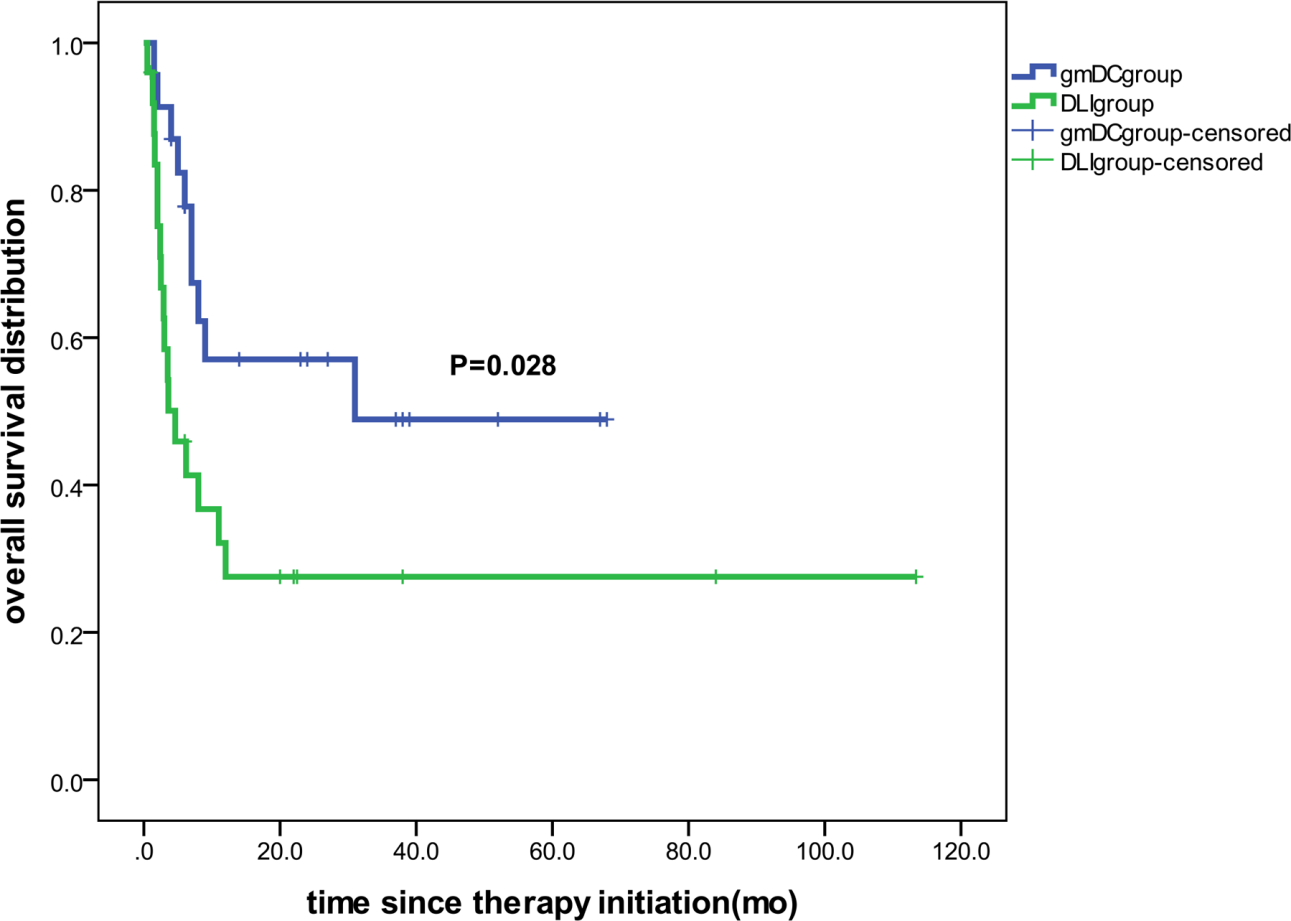
Kaplan-Meier estimates of overall survival. The 3-year probability of OS was significantly higher in the Ad-siSSF-DC group (*n* = 23) than in the DLI group (*n* = 25) (48.9 % vs. 27.5%, respectively). *P* = 0.028, as determined by log-rank test.

Relapse after allo-HSCT is the leading cause of treatment failure for AL patients. Thus, we conducted a 2-phase clinical trial, in which we first assessed 48 AL patients with relapse after allo-HSCT to analyze the efficacy of Ad-siSSF-DC treatment compared with DLI by observing OS and incidence of GVHD. The overall study design is shown in Figure 4, and patient characteristics are summarized in Table 1. Patients were randomized to either the Ad-siSSF-DC (*n* = 23) or DLI group (*n* = 25), and the results are summarized in Table 2. We found that the 3-year OS in the Ad-siSSF-DC group was 48.9% compared with 27.5% in the DLI group (*P* = 0.028) (Figure 5). Moreover, the CR rate was higher in the Ad-siSSF-DC group (13 of 23, 57%) compared with the DLI group (12 of 25, 48%), although this difference was not statistically significant (*P* = 0.729). Overall acute and chronic GVHD (aGVHD and cGVHD, respectively) rates were not statistically different: aGVHD occurred in 6 of 23 patients in the Ad-siSSF-DC group and in 11 of 25 patients in the DLI group (26% vs. 44%; *P* = 0.195). However, none of the patients in the Ad-siSSF-DC group developed grade 3 or 4 aGVHD, whereas 9 patients developed grade 3 or 4 aGVHD in the DLI group (0% vs. 36%; *P* = 0.001), suggesting that Ad-siSSF-DCs are a safe alternative approach to treat AL relapse.

### Autologous Ad-siSSF-DCs are safe and efficacious in AML patients.

**Figure 6:**
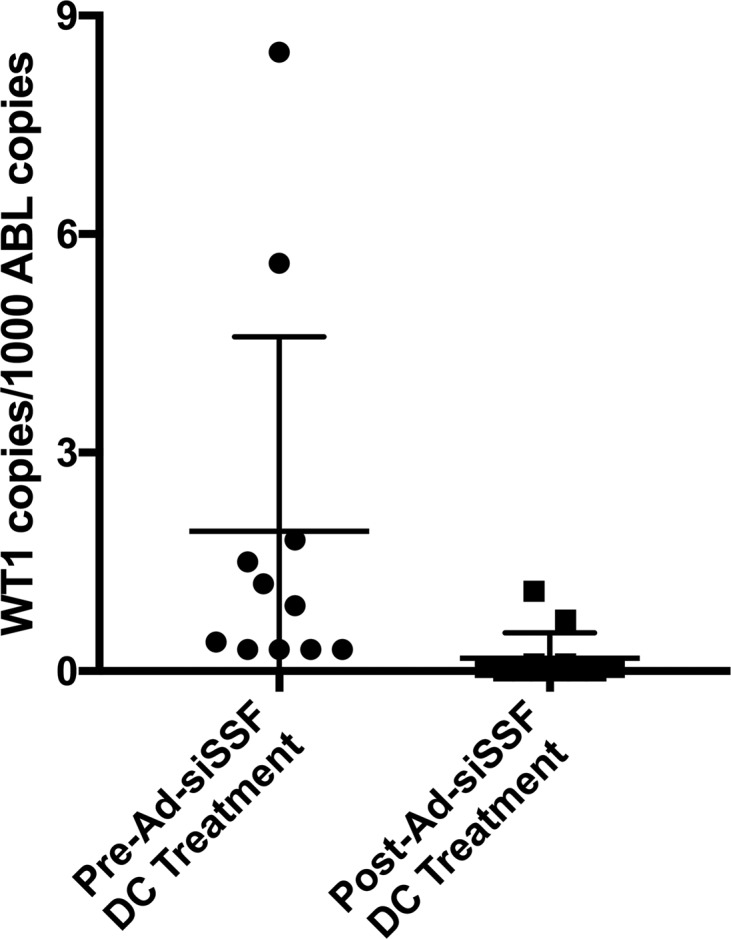
Comparing WT1 expression in AL patients before and after treatment. For the majority of the patients (*n* = 12), WT1 expression decreased drastically after infusion of Ad-siSSF-DCs. Across all patients, the pretreatment mean of WT1 copies was 1.92 per 1,000 ABL copies versus 0.18 WT1 copies detected after treatment. The complete remission rate was 83%. The GVHD rate was 50%, and no cases of grade 3 or grade 4 GVHD were observed.

The second phase of our clinical trial analyzed the clinical efficacy and adverse events associated with Ad-siSSF-DC treatment for early molecular relapse after allo-HSCT in 12 AML patients (patient characteristics are summarized in Table 3). To monitor minimal residual disease, we used quantitative reverse transcription–PCR (qRT-PCR) to quantify Wilms’ tumor gene (WT1) expression. WT1 is a transcription factor that is overexpressed in the cancer cells of most AML patients and can be measured in both bone marrow and peripheral blood samples (45). This analysis showed that WT1 expression was undetectable in 83% (10 of 12) of patients after Ad-siSSF-DC treatment, indicating CR, such that mean WT1 expression was significantly decreased after treatment (*P* = 0.031; Figure 6). The aGVHD rate was 50% (6 of 12), and again no grade 3 or grade 4 aGVHD was observed. These clinical results suggest that Ad-siSSF-DCs represent an effective immunotherapy for patients with relapsed AML after allo-HSCT.

## Discussion

The results of this study demonstrate that hDCs transduced with Ad-siSSF to coexpress SOCS1 shRNA, a survivin-MUC1 fusion protein, and secretory flagellin possess potent immunostimulatory activity to induce survivin- and MUC1-specific CTLs. The enhanced immunopotency of Ad-siSSF–transduced DCs is likely due to increased surface expression of costimulatory and antigen presentation molecules and enhanced production of proinflammatory cytokines and chemokines. Ad-siSSF–transduced DCs also expressed obviously higher levels of CCR7, a key lymph node–homing receptor, and more efficiently migrated toward CCR7 ligands, suggesting that these DCs may have an enhanced migratory ability to draining lymph nodes in immunized patients. These promising in vitro results prompted us to translate Ad-siSSF into clinical studies as a DC vaccine platform with powerful immunopotency against broadly expressed TAAs.

The inhibition of SOCS1 alone does not trigger proinflammatory signaling, and TLR5stimulation alone is inefficient to break self-tolerance to stimulate effective antitumor responses (14, 17). However, the combined inhibition of SOCS1 and TLR stimulation activated DCs with unbridled signaling of the JAK/STAT and TLR signaling pathways to persistently induce antigen-specific CTL responses, resulting in the breakage of self-tolerance at the host level and effective antitumor immunity in mice (14, 17). There is a risk of autoimmune toxicities associated with inhibition of SOCS1, as SOCS1-deficient mice die within 3 weeks of birth due to severe inflammation in many organs and tissues (46, 47), and the transfer of SOCS1-deficient bone marrow cells into lethally irradiated mice results in lethal inflammation (46, 48). However, the risk of autoimmune damage induced by SOCS1-downregulated DCs may be limited and manageable. Additionally, inhibiting SOCS1 in DCs may be safer than systemically targeting other immune checkpoints, such as CTLA4, since negative regulators in the CTLs of immunized patients would remain functional.

We selected flagellin, a TLR5 agonist, for our DC vaccine over other immune stimuli based on several considerations. First, DCs are known to use TLRs to recognize conserved pathogen-associated molecular patterns, such as LPS, flagellin, unmethylated bacterial DNA (CpG), and dsRNA (49–51). TLR activation then promotes DC maturation by activating NF-κB and MAP kinase signaling cascades to mediate the upregulation of hundreds of proinflammatory genes (49–51). To our knowledge, flagellin is the only established TLR agonist that can be encoded by an expression vector for DC transduction. Additionally, because TLR5 signaling in DCs requires cell surface interaction with flagellin, the flagellin gene in the Ad-siSSF vector was modified to contain a signal leader sequence, allowing for its secretion and subsequent binding to surface TLR5 on transduced DCs in an autocrine or paracrine manner in order to maximize its stimulatory activity.

Increasing numbers of TAAs have been identified for tumor vaccination. An ideal TAA should be highly expressed on tumor cells but not on normal cells. Survivin has been found to be highly expressed in the majority of human cancers, including human leukemia cells, but is largely undetectable in most normal tissues (52, 53). Moreover, in an analysis of human transcriptomes, survivin was identified as the fourth highest differentially expressed gene in human cancer tissues, including human leukemia cells (53), compared with normal tissues (36). Furthermore, MHC-restricted survivin epitopes have been identified, and several clinical trials have demonstrated the immunogenicity of survivin to induce antitumor immune responses in mice and cancer patients (52, 54–56). Thus, survivin was selected as an immunogen for the induction of antitumor immunity in our vaccine.

It is generally believed that targeting multiple TAAs may be more effective than targeting one TAA, and MUC1 was selected as a second immunogen for our DC vaccine. MUC1 is a highly glycosylated transmembrane protein with an extracellular tandem repeat domain (TRD) that is normally expressed on the apical surface of ductal epithelia (57). MUC1 is widely overexpressed in various cancers, including hematological malignancies (58), often in abnormal hypoglycosylated forms with exposed T cell epitope (59). Moreover, the MHC-restricted immunodominant epitopes within the TRD have been characterized. Immunization with MUC1, antigenic peptides, or pulsed DCs can induce MUC1-specific CTL responses in mice and human cancers (60–63). Importantly, in contrast to intracellular survivin, MUC1 is expressed on the surface of tumor cells and can be recognized by antibodies for antibody-mediated tumor destruction. Because of these properties, our vaccine targeting survivin and MUC1 might be broadly applicable for the treatment of various types of human cancer.

DCs can be manipulated ex vivo and applied as a vaccine to induce tumor-specific CTLs and immunological memory that function to suppress tumor relapse (64). Recently, ectopic expression of TAAs in gmDCs was shown to enhance the efficiency of DC therapeutic vaccination to induce immunity against malignancies (65). Genetic modification of DCs with TAA genes allows for stable TAA expression and the presentation of multiple and cryptic epitopes by both MHC class I and class II molecules (66). In the current study, DCs were genetically modified to express survivin and MUC1 TAAs in addition to SOCS1 shRNA and flagellin, and these gmDCs were generally well tolerated clinically and capable of inducing TAA-specific immunity.

Disease recurrence remains a major challenge for the clinical treatment of AL. Multiple patterns of relapse can be distinguished, including most frequently hematological relapse and molecular relapse (67), and treatment options for patients with relapsed AL after allo-HSCT are limited. Withdrawal of immunosuppressive drugs is usually the first measure, and this by itself can control leukemia in a limited number of patients. In cases of nonresponse and for patients suffering disease recurrence after discontinuation of immunosuppressive agents, DLI or second HSCT may be considered. Response to DLI requires a critical number of T cells and correlates with a high risk of GVHD, and, due to complications and relapse, the overall response of most patients to DLI is poor. In the first stage of the present study, an open-label, controlled trial for AL showed that infusion of Ad-siSSF-DCs was potentially useful for AL patients with relapse after allo-HSCT. This effect was evident in terms of both OS and CR. Importantly, patients treated with Ad-siSSF-DCs had significantly fewer grade 3 and 4 GVHDs compared with the DLI group. This finding is critical because DLI therapy is usually associated with serious toxicities; thus, Ad-siSSF-DCs are a safe alternative approach to current standard treatments. Because a limited number of ALL patients were enrolled in the first phase of the trial, the results may not be generalizable to other types of leukemia, and further trials are warranted. The second phase of the clinical study was an open-label, noncontrolled trial that demonstrated that infusion of Ad-siSSF-DCs was potentially useful for AML patients with early molecular relapse after allo-HSCT, which was evident in terms of CR (83%). Importantly, patients treated with Ad-siSSF-DCs had no grade 3 or 4 GVHD, again indicating that treatment with Ad-siSSF-DCs is likely safe. These results designate patients with molecular relapse as candidates for this treatment strategy.

Our study did have some limitations that are worth noting. First, we did not analyze the persistence of Ad-siSSF in monkeys beyond 14 days. However, both a lack of toxicity and robust T cell responses were observed at 14 days, signifying a lasting immune response. In addition, we did not use double-blind approaches, which may have caused bias in our evaluation of certain outcomes, such as GVHD, in the clinical studies. The small numbers of patients limited statistical power, thereby potentially obscuring statistically significant differences. Finally, a contribution of coadministered cytokine-induced killer cells to the observed vaccine efficacy cannot be ruled out. Notwithstanding these limitations, our findings indicate that Ad-siSSF is a potent activator of DCs and that Ad-siSSF-DCs may be a safe and effective treatment for AML patients with early molecular relapse after allo-HSCT. Larger long-term clinical studies are warranted to assess the generalizability of these findings and to deepen our understanding of this promising therapeutic approach.

## Methods

### Study design, participants, and treatments.

This study included open-label, controlled and noncontrolled trials. Forty-eight patients with hematopoietic relapsed AL after allo-HSCT and 12 AML patients with early molecular relapse after allo-HSCT were enrolled from July 1, 2009, to July 1, 2014, for the first and second phases of the trial, respectively, at the Affiliated Hospital of Academy of Military Medical Sciences. Inclusion criteria were expected survival duration of more than 3 months and age of between 8 and 60 years. Patients with autoimmune diseases, HIV infection, or chronic active hepatitis were excluded. This trial is registered with ClinicalTrials.gov (NCT01956630).

AML patients received a standard-dose chemotherapy regimen of cytarabine plus daunorubicin (DA) or cytarabine plus mitoxantrone (MA), and ALL patients received a regimen of VDCP or VDLP (V, vincristine; D, daunorubicin; C, cyclophosphane; P, prednisone; L, L-asparaginase). All patients underwent a conditioning regimen of cyclophosphamide and total body irradiation. Patients received 4 s.c. injections of 2 × 10^7^ to 5 × 10^7^ Ad-siSSF-DCs in the groin, axilla, and neck on days 7, 9, 11, and 13 in each cycle and 2 subsequent infusions of cytokine-induced killer cells (>10^9^) (68) after Ad-siSSF-DC treatment. WT1 was monitored in blood and bone marrow cells by qRT-PCR (Supplemental Table 1), where values above 2 and 25 copies of WT1 mRNA per 1,000 ABL copies in blood and marrow, respectively, were considered to be above normal background and, thus, indicative of residual disease. The cycle was repeated until WT1 became undetectable by qRT-PCR or GVHD appeared. Alternatively, patients received i.v. DLI at doses of 2 × 10^7^, 5 × 10^7^, and 1 × 10^8^ CD3^+^ cells at months 1, 2, and 3, respectively, unless WT1 became undetectable by qRT-PCR or GVHD appeared.

### Outcome measures and follow-up.

The median follow-up time for the 48 AL and 12 AML patients was 23 months (range, 3–60 months) and 16 months (range, 6–39 months), respectively. No patients were lost to follow-up. The primary outcome measures in were CR and OS in the AL patients or CR and GVHD in the AML patients. CR was defined as recovery of hematopoiesis, with an absolute neutrophil count above 1.5 × 10^9^/l, a platelet count above 100 × 10^9^/l, normalization of marrow blasts (5%), and negative expression of WT1. OS refers to the time from diagnosis (relapse of AL after allo-HSCT) to either death or the last day of follow-up, July 1, 2014. GVHD was defined according to published criteria (69).

### Monkey study.

The monkey study was conducted in compliance with Good Laboratory Practice regulations. Groups of naive female rhesus monkeys (~3 years old, *n* = 3 per group) were intramuscularly injected once with the recombinant Ad-siSSF vector at a dose of 2 × 10^12^ vp/kg or control PBS. Monkeys were necropsied on day 14 after injection to evaluate acute toxicity and immune responses. Tissues and organs were collected, processed to slides, stained with hematoxylin and eosin, and examined microscopically.

### Generation of recombinant replication-defective adenoviruses.

An Ad-Easy system (E1 and E3 deletion; Quantum Biotechnologies Inc.) was used to construct and generate replication-defective adenoviruses, as described previously (70) and in the Supplemental Methods. Specifically, an Ad5 vector encoding SOCS1 shRNA, survivin, MUC1, and flagellin sequence fragments was constructed in our lab. A series of experiments, such as a pilot-plant test, acute toxicity test, and safety evaluation test, were completed before approval for clinical application by the Medical Department of Central Logistics Department in 2009. The clinical grade good manufacturing practice (GMP) Ad-siSSF vector was produced and tested by Shenzhen Tsinghua Yuanxin Biotechnology Inc. (Shenzhen, China). The criteria for lot release of the GMP Ad-siSSF vector included a negative general sterility test, absence of mycoplasma, and a virus particle (vp)/infectious unit ratio ranging from 10:1 to 40:1.

### In vitro studies.

Prior to in vivo studies in monkeys or humans, multiple in vitro assays were conducted to evaluate the effects of Ad-siSSF-DCs on antigen presentation and tumor-specific CTL responses. First, the phenotypes of PBMC-derived DCs and T cells were determined by flow cytometric analyses. FITC-, PE-, and APC–conjugated mAbs against human CD40 (HB14; Biolegend), CD80 (L307.4; BD Biosciences), CD86 (2331; BD Biosciences), OX40L (ANC10G1; LifeSpan Biosciences), MHC II (HLA-DR, TU39; BD Biosciences), TLR5 (624915; R&D Systems), GITRL (109101; R&D Systems), and CCR7 receptor (150503; R&D Systems) and matched isotype controls were used for multiple color staining of DCs. FITC- or PE-conjugated mAbs against human CD4 (RPA-T4), CD8 (G42-8), and IFNG (4S.B3) (BD Biosciences) were used to stain T cells. The following methods were used to further characterize the activity of Ad-siSSF-DCs: ELISA and cytokine antibody arrays were used to measure cytokine and chemokine concentrations in Ad-siSSF DC cultures (IFNG, IL6, IL12 p40/p70, TNFA, and RANTES ELISA kits purchased from BD Biosciences); the migratory ability of Ad-siSSF-DCs was assessed using a standard Transwell in vitro migration assay (CCL21 antibody purchased from GeneTex); the endocytotic capability of Ad-siSSF DCs was tested using dextran–Texas red (Invitrogen); the immunopotency of Ad-siSSF-DCs to prime antigen-specific CTLs was evaluated using in vitro T cell immunization assays (rhIL2, rhIL4, and rhGM-CSF purchased from R&D Systems); [^3^H] thymidine incorporation and CSFE (Invitrogen) labeling were used to assess the effect of Ad-siSSF-DCs on Treg proliferation and suppression; IFNG (mAb 1-D1K; Mabtech) ELISpot assays were used to determine the number of antigen-specific T cells in cocultures of DCs and autologous T cells; the cytolytic activity of primed T cells to human tumor cells (MCF7, A-498, SK-OV-3, and MDA-MB-231; ATCC) was evaluated using a standard ^51^Cr release assay; Western blot analyses were used to assess SOCS1 downregulation by shRNA as well as the expression of survivin-MUC1 fusion protein and flagellin following Ad-siSSF transduction; and qRT-PCR was used to evaluate the relative expression of human SOCS1 in hDCs. Detailed protocols for each assay can be found in the Supplemental Methods and supporting references.

### Statistics.

OS was calculated from the initiation of treatment (relapse of AL after allo-HSCT) to death, and living patients were censored at the time of last contact. The SPSS 16.0 software package (SPSS Inc.) was used for all statistical analyses. Survival data were analyzed by the log-rank test, and survival curves were assessed using the Kaplan-Meier method. Student’s 2-tailed *t* tests were used to compare differences between groups, where *P* < 0.05 with a 95% confidence limit was considered statistically significant. Results are typically presented as mean ± SEM.

### Study approval.

This study complies with the Declaration of Helsinki. The clinical study was approved by the ethics committee of the Affiliated Hospital of Academy of Military Medical Sciences. All participants gave written informed consent prior to participation in the study. Rhesus monkeys were obtained from Chengdu Huaxi Haiqi Medical Technology Co. Ltd./ National Chengdu New Drug Safety Evaluation Center, treatment was in accordance with the regulations outlined in the US Department of Agriculture Animal Welfare Act (9 CFR, parts 1–3), and conditions were specified in the *Guide for the Care and Use of Laboratory Animals* (National Academies Press, 2011). The monkey study protocol was approved by the Chengdu Biological Safety Evaluation Center’s IACUC (Sichuan, China).

## Author contributions

XFH, BH, XTS, LR, LJ, and SYC designed and generated the vaccine and performed the preclinical studies. DW, LH, MJ, BZ, HN, YL, CX, XL, BL, ZY, JH, JC, FY, HG, GD, LL, and HC designed and performed the clinical studies. SYC, HC, LH, MJ, BZ, LL, and LJ analyzed data and wrote the manuscript. LL provided statistical analyses.

## Supplementary Material

Supplemental data

ICMJE disclosure forms

## Figures and Tables

**Table 3:**
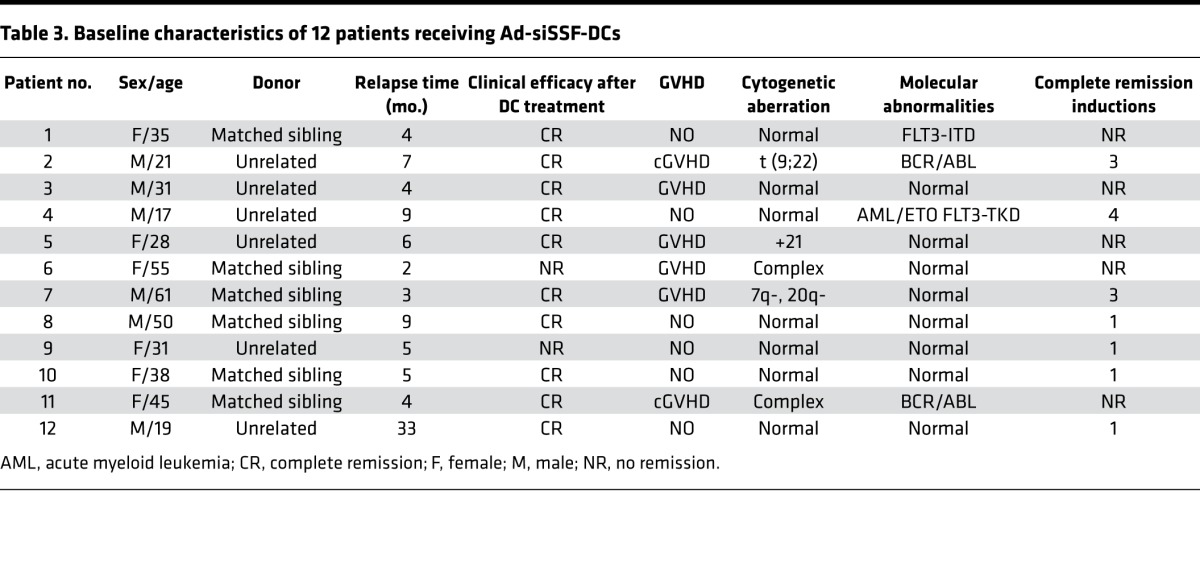
Baseline characteristics of 12 patients receiving Ad-siSSF-DCs

**Table 2:**
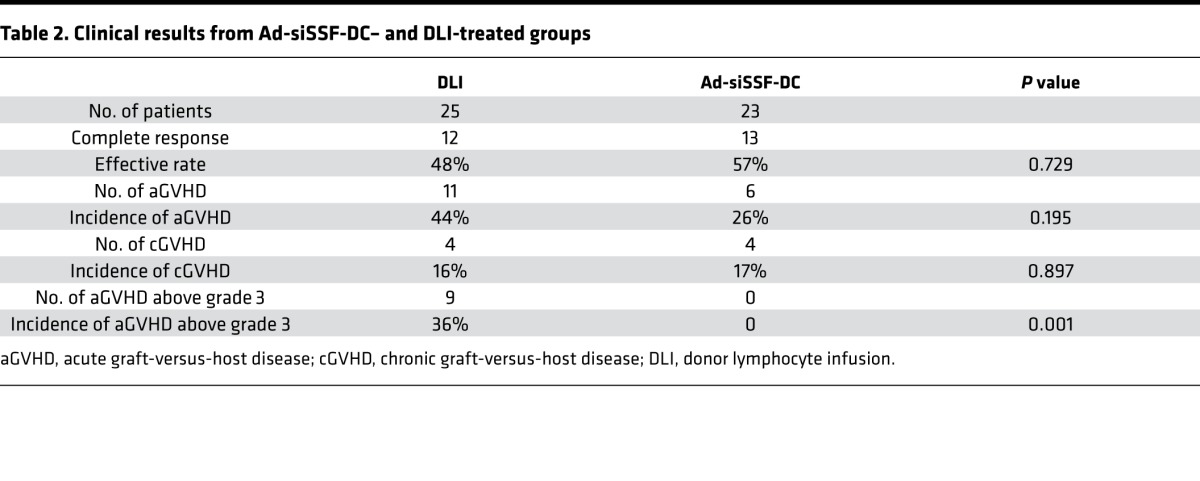
Clinical results from Ad-siSSF-DC– and DLI-treated groups

**Table 1:**
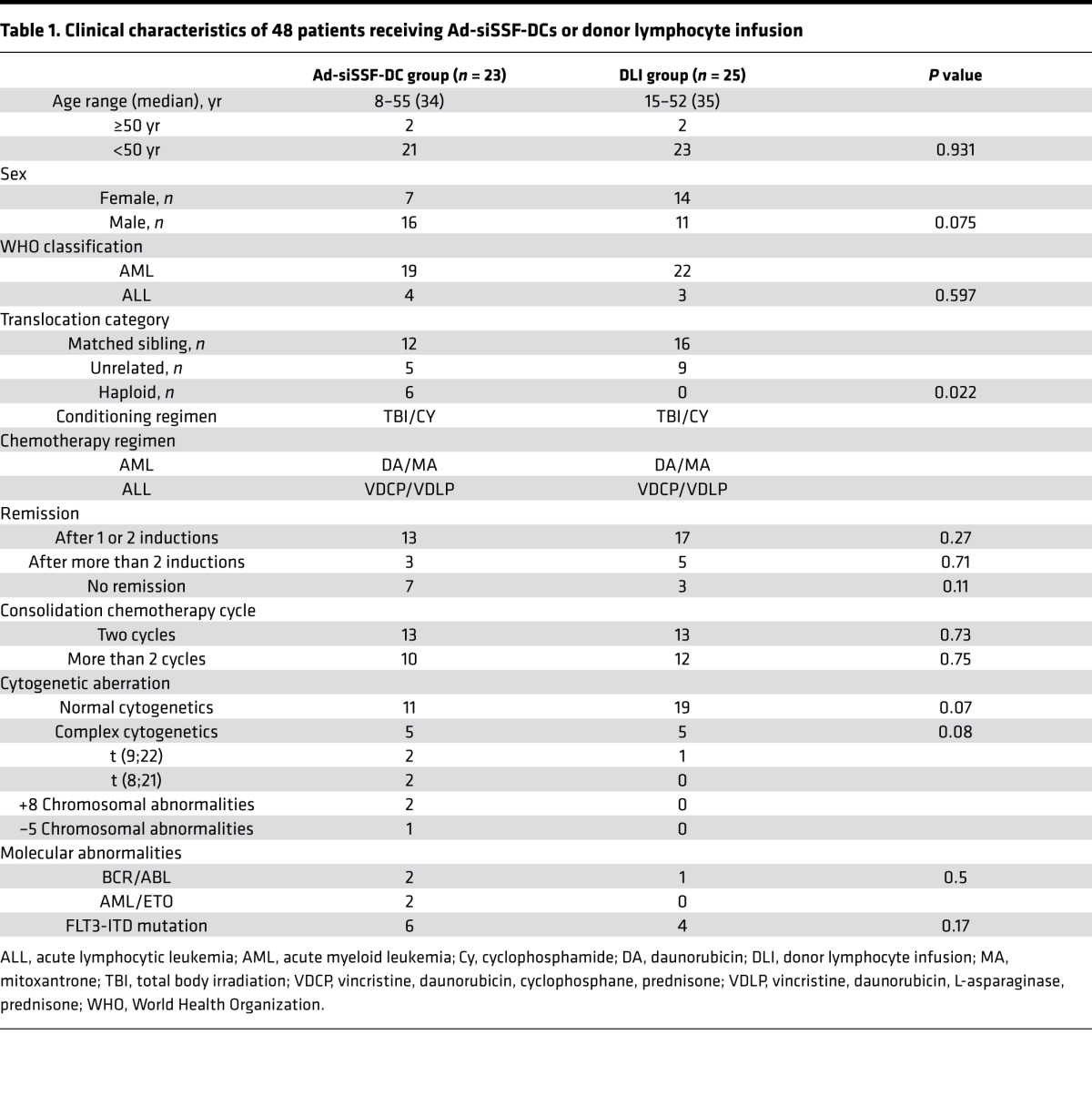
Clinical characteristics of 48 patients receiving Ad-siSSF-DCs or donor lymphocyte infusion

## Version 1 02/08/2018

Electronic publication
