# Supplementary material for: Efficacy of intracellular immune checkpoint-silenced DC vaccine
Source: JCI Insight. 2018 Feb 8;3(3):e98368. doi: 10.1172/jci.insight.98368 (PMC5821183; doi:10.1172/jci.insight.98368)
Supplement: Supplemental data [file jciinsight-3-98368-s001.pdf]

Efficacy of Intracellular Immune Checkpoint-SilencedDendritic Cell Vaccine

Danhong Wang, Xue F. Huang, Bangxing Hong, Xiao-Tong Song, Liangding Hu, Min Jiang, Bin Zhang, Hongmei Ning,Yuhang Li, Chen Xu, Xiao Lou, Botao Li, Zhiyong Yu,Jiangwei Hu, Jianlin Chen, Fan Yang, Haiyan Gao, Guoliang Ding, Lianming Liao, Lisa Rollins, Lindsey Jones, Si-Yi Chen ,Hu Chen

Supplementary Materials and Methods

Design and production of recombinant adenoviral vector Ad-siSSF

In order to construct a recombinant replication-deficient adenoviral vector, designated as Ad-siSSF, which coexpresses a small hairpin human SOCS1-siRNA (siSOCS1), dominant negative (DN) survivin and MUC1 fusion protein, and a secretory flagellin, the TLR5 ligand (**SupplementaryFig. 1A**), we first modified survivin, MUC1, and flagellin genes, as well as identified siRNA that can effectively silence human SOCS1 (5'-CACGCACTTCCGCACATTC-3'). Because of the anti-apoptotic biological activity of survivin for the potential promotion of malignant cell survival and transformation, two mutations were introduced into the survivin gene to generate a DN survivin mutant. A substitution mutation of Thr with Ala in position 34 was made to eliminate the $p34^{cdc2}$-cyclin B1 phosphorylation site and anti-apoptotic activity, as described previously(1). Moreover, to avoid possible reverse mutation of the survivin mutant back to a functional phosphorylation site, a 16 amino acid truncation at the N-terminus of survivin was further made since the N-terminal sequence is required for its inhibition of apoptosis (2). We used a short human mucin 1 (MUC1) fragment containing only a three 20 amino acid tandem repeat sequence, because a known dominant T cell epitope is located within the 20 amino acid repeat(3)and full-length or lengthyMUC1 protein was found to have immune suppressive activity by inhibiting a Th1-type CTL response(4). To generate a DN survivin-MUC1 fusion gene (DN SM) as target tumor-associated antigens for the Ad-siSSF vector, the double mutated DN survivin was genetically linked in-frame to the three 20 amino acid repeat fragment of MUC1. For facilitating the secretion and subsequent interaction with surface TLR5 on DCs of flagellin by transfected DCs, a signal leader sequence derived from human tyrosinase was genetically linked to the N-terminus of the flagellin gene. We also identified siRNA with the ability to specifically downregulate human SOCS1 (**SupplementaryFig. 1B**).

The resultant Ad vector Ad-siSSF, plus Ad-SM that only expresses the DN SM fusion protein and Ad-GFP were constructed, as schematically shown in **SupplementaryFig. 1A**, and insertion of these genes in the recombinant adenovirus was confirmed by DNA sequencing. The ability of the Ad-siSSF virus to efficiently coexpress the three components of siS1, DN SM fusion protein, and secretory flagellin in transfected cells was demonstrated by Western blotting analysis (**SupplementaryFig. 1C**).

Generation of recombinant replication-defective adenoviruses

An Ad-Easy system (E1 and E3 deletion; Quantum Biotechnologies Inc., Palo Alto, CA) was used to construct and generate replication-defective adenoviruses, as described previously (5). The DN truncated human survivin mutant gene was generated by PCR using the Thr34-Ala mutant hSVNT34A(6)as a template with a pair of primers (**SupplementaryTable 1**). Oligonucleotide duplexes containing the human MUC1 three-repeat MUC1 sequence were synthesized by Operon and PCR-amplified with primers (**SupplementaryTable 1**) to add cloning sites HindIII and NotI. The amplified survivin and MUC1 fragments were digested with restriction enzyme HindIII and ligated at room temperature for 1 hr. The DN survivin mutant-MUC1 fusion fragment was then amplified by PCR to generate the DN survivin mutant-MUC1 fragment containing a SalI restriction site at the 5' end and a NotI site at the 3' end. The modified flagellin (fliC) gene with a signal leader sequence from human tyrosinase was amplified by PCR using *Salmonella entericaserovar Typhimurium* (ATCC, Manassas, VA) as a template with a pair of primers (**SupplementaryTable 1**). Encephalomyocarditis virus IRES sequence was amplified from the pShuttle-CMV-HSP-IRES-E1A vector generated in our previous study (7) with [illegible]

using *Salmonella entericaserovar Typhimurium* (ATCC, Manassas, VA) as a template with a pair of primers (**SupplementaryTable 1**). Encephalomyocarditis virus IRES sequence was amplified from the pShuttle-CMV-HSP-IRES-E1A vector generated in our previous study(7)with primers (**SupplementaryTable 1**). Survivin-mucin-IRES fragment was constructed by ligation of NotI digested surviving-mucin fragment and IRES fragment. The resultant shuttle vector pshuttle-siSSF was constructed by inserting SalI/Xhol-digested survivin mutant-MUC1-IRES fragment into a SalI/Xhol-digested pshuttle-SF vector, which was previously constructed by inserting the modified, secretory flagellin into an XhoI/Xbal-digested pshuttle-hSOCS1 vector. The pshuttle-hSOCS1 vector was generated by inserting the human H1 RNA promoter, human SOCS1-siRNA and transcriptional termination sequence PCR-amplified from the pSuper-hsiS1 vector into an Ad pshuttle vector. Subsequently, the recombinant replication-deficient Ad-siSSF virus containing the human SOCS1 siRNA under H1 RNA promoter (from pSuper vector), and DN human surviving mutant-MUC fusion gene linked to the modified flagellin with the encephalomyocarditis virus IRES sequence under the control of CMV promoter was generated according to the manufacturer's instruction. The insertion of these gene fragments in the recombinant Ad virus was confirmed by PCR and DNA sequencing (**SupplementaryFig. 1B**). Recombinant adenoviruses were produced and titrated in 293 cells according to the manufacturer's instructions (Quantum Biotechnologies Inc.).

Generation of human DCs and T cells from peripheral blood mononuclear cells (PBMC) and virus transfection

Manufacture of the genetically modified DCs was performed at the cGMP facility at the Affiliated Hospital of Academy of Military Medical Sciences, Beijing, China.A single unmobilizedleukapheresis processing 1 plasma volume was performed to obtain peripheral blood mononuclear cells (PBMCs). PBMCs were isolated by Ficoll-paque (GIBCO-BRL, Grand Island, NY) density gradient centrifugation of heparinized blood obtained from buffy coat preparations of healthy donors. Cells in the culture medium were seeded into 150-mm cell culture plates at the cell density of 5×10^6/ml. After 3 hrs of incubation at 37°C, nonadherent cells were removed and frozen at 1×10^6/ml for future use. The adherent blood monocytes were cultured in CellGro DC culture medium (Freiburgim Breisgau, Germany) supplemented with human recombinant granulocyte-macrophage colony-stimulatory factor (rhGM-CSF, 1,000 U/ml; R&D Systems,Minneapolis, MN) and rhIL4 (1,000 U/ml; R&D Systems).rhGM-CSF and rhIL4 were replenished every 3 days. On day 5 PBMC-derived DCs were harvested, reseeded onto 12-well culture plates at the cell density of 1×10^6/ml, and then transfected with different MOIs of Ad vectors in serum-free RPMI1640 medium for 1.5 hr, followed by incubation in DC culture medium supplemented with 1000 U/ml rhIL4 and 1000U/ml rhGM-CSF for 2 days. Ad-transfected DCs were harvested and washed with serum-free RPMI1640 medium for further studies. T cells were generated in vitro from non-adherent PBLs with soluble anti-CD3 antibody (BioLegend, San Diego, CA) in the presence of 300 iU of recombinant IL2 (R&D Systems). The leukapheresis cell product and all the reagents used for vaccine preparation were tested for mycoplasma, endotoxin, bacterial and fungal sterility. The final product, only when confirmed to be free of contaminants, was administered to the research subjects.

Flow cytometric analysis of human PBMC-derived DCs and T cells

Phenotypes of DCs and T cells were determined by flow cytometric assays. Fluorescein isothiocyanate (FITC)-, phycoerythrin (PE)- and (APC)-conjugated monoclonal antibodies (Mabs) against human CD40 (B-ly6), CD80 (L307.4), CD86 (2331), HLA-DR (TU39), HLA-A2 (BB7.2), CD11c (B-ly6) (BD Biosciences), OX40 ligand (OX40L) (ANC10G1), GITR ligand (GITRL) (109101), and CCR7 receptor (150503) (R&D Systems), and matched isotype controls were used for multiple color staining of DCs. FITC- or PE- conjugated MAbs against human CD4 (RPA-T4), CD8 (G42-8), and IFNG (4S.B3) (BD Biosciences) were used to stain T cells. DC viability was determined with anti-Annexin V staining (BD Biosciences). Mouse anti-human CD227 (MUC1, SeroTec, CloneC595) were used to stain human tumor cell lines. Cells were washed and suspended in PBS containing 2% FCS and 0.02% sodium azide. For direct staining, cells were pre-incubated in 200 μg/ml of polyclonal human IgG (Sigma-Aldrich, St. Louis, MO) for 10 minutes on ice. Specific labeled Mabs or appropriate isotype controls were added, and cells were further incubated on ice for 25 minutes. For indirect staining, cells were preincubated with 200 μg/ml of polyclonal human IgG before incubation with anti-human CD227 Ab. Specific Mab was revealed with goat anti-mouse Ab labeled with FITC (Santa Cruz Biotechnology, Santa

cells were further incubated on ice for 25 minutes. For indirect staining, cells were preincubated with 200 μg/ml of polyclonal human IgG before incubation with anti-human CD227 Ab. Specific Mab was revealed with goat anti-mouse Ab labeled with FITC (Santa Cruz Biotechnology, Santa Cruz, CA). Staining with goat anti-mouse Ab labeled with FITC without primary antibodies was always performed in a separate tube as control. Stained cells were then analyzed on a FACSAria (Becton Dickinson) with FloJo software. The purity of the cultured DCs and T cells was determined to be greater than 80%.

ELISA and cytokine antibody array assays

Cytokine and chemokine concentrations in DC cultures were measured by commercially available two-site sandwich enzyme-linked immunosorbent assays (ELISA) from BD Biosciences (IL6, IL12, IL10, TNFAa, IFNGgand RANTES) according to manufacturers' instructions.(8)RayBio Human Cytokine Antibody Array 3 (Ray Biotech, Norcross, GA) was used to detect the relative levels of 42 human cytokines and chemokines in the culture media of DCs according to the manufacturer's instruction.

Chemotaxis assay

DC migration was measured using a transwell system(24-well plates; 8 ìM pore size; Costar, Corning, NY)(9).Human PBMC-derived DCs transfected with different Ad vectors for 48 hr were collected and seeded into 24-well transwell plates (5 ìM pore size, vendor) in upper chambers (100 μl of 1×10^5 cells/ well). 500 μl DC culture medium with 100 ng/ml CCL21 (GeneTex, Irvine, CA) or only culture medium was plated in the lower chambers. Wells with medium only wereused as a control for spontaneous migration. After culture for 3 hr at 37ºC, 500 μl medium in the lower well was collected and cell numbers were calculated with FACS collection for 50 seconds. The counts fell within a linearrange of the control titration curves obtained by testing increasingconcentrations of cells. The mean number of spontaneously migratedcells was subtracted from the total number of cells that migratedin response to the chemokine. Migration rate (%) = (cell number in CCL21 medium- cell number in DC medium)/(cell number in DC medium) × 100.

Endocytotic assay of human DCs

The endocytotic capability of PBMC-derived DCs was tested using Dextran-Texas Red, as previously reported (10). 1×10^6 cells human PBMC-derived DCs were transfected with different Ad vectors at an MOI of 25,000 viral particles (vp). 48 hrs later, 2×10^5 transfected DCs were pulsed with Dextran-Texas Red (Invitrogen, Carlsbad, CA)at a concentration of 1 mg/ml for 4 hrs. Cold staining buffer (1% FBS in PBS) was added to stop the reaction. The cells were washed four times and stained with PE-conjugated anti-CD11c Abs, and then analyzed on a FACSAria. Nonspecific binding of dextran to DCs, determined by incubation of DCs with Dextran-Texas Red at 4°C, was shown. The medium used in the culture was supplemented with GM-CSF, because the ability of DCs to capture Ag is lost if DCs were cultured without GM-CSF(11).

In vitro T cell sensitization

In vitrosensitization of autologous T cells was performed as reported(12-14). 1×10^6 human PBMC-derived DCs were mock-transfected or transfected with Ad vector at an MOI of 25,000 vp for 1.5 hr in 1ml serum-free RPMI1640 medium in 12-well culture plate, followed by the addition of 1ml DC culture medium with 20% FBS for 48 hrs. Transfected DCs were harvested, washed with RPMI1640, and then cocultured with autologous T cells at a DC:effector ratio of 1:20 in 2 ml DC culture medium without rhIL4 and rhGM-CSF at 37 °C in a humidified atmosphere containing 5% CO_2 for 7 days. Every 3 days, the cultures were replenished with recombinant human IL2 (R&D Systems) at a concentration of 50 U/ml. The T cells were restimulated weekly with freshly prepared mock or Ad-transfected autologous DCs. After 14 days of cocultures, the T cells were collected for intracellular cytokine staining, ELISPOT and CTL assays.

In vitro T cell proliferation and suppression assays

To assess the effect of Ad-transfected DCs on Treg function, human PBMC-derived DCs (day 5) were transfected with different Ad vectors at an MOI 25,000 vp for 48 hrs as described above. Human $CD4^+CD25^+$Treg cells and $CD4^+CD25^-$Teff cells from different donors were isolated by using human $CD4^+CD25^+$ MACS kit (MiltenyiBiotec, BergischGladbach, Germany) as specified. 5×10^5 Ad-transfected DCs were cocultured with 1×10^5 autologous $CD4^+CD25^+$Treg cells for 5

Human CD4$^+$CD25$^+$Treg cells and CD4$^+$CD25$^-$Teff cells from different donors were isolated by using human CD4$^+$CD25$^+$ MACS kit (MiltenyiBiotec, BergischGladbach, Germany) as specified. 5×10^5 Ad-transfected DCs were cocultured with 1×10^5 autologous CD4$^+$CD25$^+$Treg cells for 5 days in 12-well cell culture plate. 1×10^5 CD4$^+$CD25$^+$Treg cells were purified with CD4$^+$CD25$^+$ MACS kit and cocultured with 1×10^5 CD4$^+$CD25$^-$Teff cells and 5×10^4 autologous mock DC in the presence of 0.5 μg/ml of anti-human CD3 for another 60 hr in 96-well plates. 1 μCi of [^{3}H] thymidine (DuPont NEU) were then added into each well and incubated for another 16 hr. The cells in triplicate wells were harvested onto fiberglass filters using Filter Mate Harvester (PerkinElmer, Waltham, MA), and filters were washed extensively. After drying, 25 μl of MicroScint 20 (PerkinElmer) were added into each well, and radioactivity was counted with a TopCount NXT Microplate Scintillation and Luminescence Counter (PerkinElmer). In parallel, 1×10^5 allogenic CD4$^+$CD25$^-$ T cells were labeled with 20 μM CFSE (Invitrogen) for 15 min at 37 °C. The CFSE-labeled T cells were added to above coculture system to monitor proliferation.

ELISPOT assays for human T cells.

ELISPOT assays were performed as described previously(13, 15),with some modifications. Briefly, MultiScreen-HA plates (Millipore, Bedford, MA) were coated overnightat 4°C with 10 μg/ml of the antihuman IFNgGmAb 1-D1K(Mabtech, Stockholm, Sweden) in coating buffer [carbonate-bicarbonatebuffer (pH 9.6)]. Plates were blocked with RPMI 1640 and 10% human serum (Bethyl, Inc., Montgomery, TX)for at least 2 hrs at 37°C. Stimulated T cellswere seeded at 2×10^5/well and cocultured with irradiated 4×10^3 survivin and MUC1 peptide-pulsedautologous DCs without Ad transfection in the presence of 20 μg/ml HLA-A2-restricted survivin peptide (ELTLGEFLKL (53)) and 20 μg/ml HLA-A2-restricted MUC1 peptide (STAPPAHGV (82,83)) at 37°C, 5% CO_2 in triplicate. Plates were incubatedat 37°C in a humidified 5% CO_2 incubator for 16 hrs, and cellswere then removed by six washes with PBS-0.05% Tween 20 (Sigma-Aldrich). Biotinylated antihuman IFNg antibody 7-B6-1 (Mabtech) at a concentrationof 1 μg/ml in PBS-0.5% human serum was added, andplates were incubated for 2 hrs at 37°C. Streptavidin-conjugatedalkaline phosphatase (Mabtech) was added for an additional hour. Cytokine-producing cells were detected after a 4-minreaction with 5-bromo-4-chloro-3-indolylphosphate and nitrobluetetrazolium (Life Technologies, Inc., Carlsbad, CA). The results were evaluatedin a blinded fashion by ZellNet Consulting, Inc. (New York,NY) with an automated ELISPOT reader system (Carl Zeiss, Inc.,Thornwood, NY), using KS ELISPOT 4.3 software.

CTL assays

CTL standard ^{51}Cr-release assay was performed, as described in our previous studies (13, 14).T cells from HLA-A2$^+$ donor-transduced DC cocultures were re-stimulated with 20 μg/ml MUC1 and 20 μg/ml survivin peptides-pulsed autologous DCs matured with 100 ng of LPS without Ad transduction for 2 hrs. Target cells (MCF7, A-498, SK-OV-3, and MDA-MB-231; ATCC, Manassas, VA) were labeled with [^{51}Cr]-sodium chromate in RPMI 1640 for 1 hr at 37 °C. Target cells (10^4) were transferred to wells of round-bottomed 96-well plates. Various numbers of DC-sensitized T cells were added to give a final volume of 200 μl and incubated for 4 hrs at 37 °C. At the end of the assay, supernatants (50 μl/well) were harvested and counted in a beta-plate counter. The percentage of cell lysis was calculated as follows: 100× ((experimental release–spontaneous release)/(maximal release–spontaneous release)). Spontaneous and maximal releases were determined in the presence of either medium or 1% SDS, respectively.

ELISPOT assays for rhesus macaque PBMCs

The rhesus macaque IFNG ELISPOT assay was performed in PBMCs.Ninety-six-well membrane-coated plates (Multiscreen-IP; Millipore) were incubated with 10 μg/ml mAb to rhesus IFNG (MD-1;U-Cytech) in 0.1 M carbonate buffer overnight. Monkey PBMCs were plated in triplicate at 2x 10^5cells/well in medium containing 10% human AB serum. The synthetic 20-mer human MUC1 peptide (HGVTSAPDTRPAPGSTAPPA), recombinant human survivin protein (Alpha Diagnostics Intl. Inc., San Antonio, TX), or irrelevant OVA protein (Sigma-Aldrich) were added to cells at the final concentration of 10 μg/ml and incubatedat 37ºC for 24 h.In addition, rhesus DCs were infected withAd-siSSF (MOI of 1000) and used as stimulators with PBMC at a ratio of 1:10.Wells were washed free of cells and then incubated with biotinylated Ab to monkey IFNG (10 μg/ml; U-Cytech) and streptavidin conjugate (Bio-Rad, Hercules, CA) before spots were developed.

[illegible]
with biotinylated Ab to monkey IFNG (10 µg/ml; U-Cytech) and streptavidin conjugate (Bio-Rad, Hercules, CA) before spots were developed.

Cell cultures

The human embryonic kidney cell line 293 (ATCC) was cultured in DMEM culture media supplemented with 10% heat-inactivated fetalbovine serum (FBS) (Invitrogen), 100 units/mL penicillin,100 µg/mL antimycotic and 100 µg/mL streptomycin at 37°C in a 5% CO_2 atmosphere. Human breast cancer cell lines, MCF-7 and MDA-MD-231, human renal cancer cell line A-498, and human ovary cancer cell line SK-OV-3 (ATCC)were maintained in RPMI 1640 media with 10% FBS, 100 units/mL penicillin,100 µg/mL antimycotic and 100 µg/mL streptomycin at 37°C.

Western blot analysis

To assess human SOCS1 downregulation by siRNA, we first used a computer program (www.dharmacon.com) to select several siRNA sequences targeting human SOCS1. We then co-transfected 293T cells with a synthetic siRNA oligonucleotide duplex (21 bp) or an irrelevant oligo duplex and a flag-tagged human SOCS1 expression vector (pCMV-hSOCS1) using GenePorter reagent(16). 48 hours after transfection, the cells were harvested and subjected to western blotting analysis. To assess the expression of survivin-MUC1 fusion protein and flagellin of Ad-siSSF vector, 293T cells were transfected with different MOIs of Ad vectors, and 48 hours later, the cells were harvested and subjected to western blotting analysis. Primary antibody goat anti-human survivin (R&D Systems), mouse anti-human MUC1 (AbDSeroTec, Raleigh, NC) and rabbit anti salmonella H:I antibody (Santens Serum Institute) were used to detect the expression levels of survivin, MUC1 and flagellin, respectively. A mouse monoclonal antibody anti-Flag (Sigma-Aldrich) was used to detect Flag-SOCS1 expression (16).

Quantitative RT-PCR analysis

The relative expression of human SOCS1 in human DCs was evaluated by quantitative real-time RT-PCR. Real-time PCR analysis was performed on an ABI 7900HT Sequence Detection System (Applied Biosystems, Foster City, CA). Pre-developed primer/probe sets for human SOCS1 from Applied Biosystems were used. SOCS1 levels were normalized to 18S rRNA, and SOCS1 expression relative to the control of mock-transfected, stimulated DCs was calculated by the Comparative DDCt method (16).

REFERENCES

1. O'Connor DS, Grossman D, Plescia J, Li F, Zhang H, Villa A, Tognin S, Marchisio PC, and Altieri DC. Regulation of apoptosis at cell division by p34cdc2 phosphorylation of survivin. *Proc Natl Acad Sci U S A*. 2000;97(24):13103-7.
2. Sun C, Cai M, Gunasekera AH, Meadows RP, Wang H, Chen J, Zhang H, Wu W, Xu N, Ng SC, et al. NMR structure and mutagenesis of the inhibitor-of-apoptosis protein XIAP. *Nature*. 1999;401(6755):818-22.
3. Fontenot JD, Mariappan SV, Catasti P, Domenech N, Finn OJ, and Gupta G. Structure of a tumor associated antigen containing a tandemly repeated immunodominant epitope. *J Biomol Struct Dyn*. 1995;13(2):245-60.
4. Carlos CA, Dong HF, Howard OM, Oppenheim JJ, Hanisch FG, and Finn OJ. Human tumor antigen MUC1 is chemotactic for immature dendritic cells and elicits maturation but does not promote Th1 type immunity. *J Immunol*. 2005;175(3):1628-35.
5. Ren W, Strube R, Zhang X, Chen SY, and Huang XF. Potent tumor-specific immunity induced by an in vivo heat shock protein-suicide gene-based tumor vaccine. *Cancer Res*. 2004;64(18):6645-51.
6. Mesri M, Wall NR, Li J, Kim RW, and Altieri DC. Cancer gene therapy using a survivin mutant adenovirus. *J Clin Invest*. 2001;108(7):981-90.
7. Huang XF, Ren W, Rollins L, Pittman P, Shah M, Shen L, Gu Q, Strube R, Hu F, and Chen SY. A broadly applicable, personalized heat shock protein-mediated oncolytic tumor vaccine. *Cancer Res*. 2003;63(21):7321-9.
8. Evel-Kabler K, Song XT, Aldrich M, Huang XF, and Chen SY. SOCS1 restricts dendritic cells' ability to break self tolerance and induce antitumor immunity by regulating IL-12 production and signaling. *J Clin Invest*. 2006;116(1):90-100.

8. [illegible]
ability to break self tolerance and induce antitumor immunity by regulating IL-12 production and signaling. *J Clin Invest*. 2006;116(1):90-100.
9. Giordano D, Magaletti DM, and Clark EA. Nitric oxide and cGMP protein kinase (cGK) regulate dendritic-cell migration toward the lymph-node-directing chemokine CCL19. *Blood*. 2006;107(4):1537-45.
10. Sallusto F, Cella M, Danieli C, and Lanzavecchia A. Dendritic cells use macropinocytosis and the mannose receptor to concentrate macromolecules in the major histocompatibility complex class II compartment: downregulation by cytokines and bacterial products. *J Exp Med*. 1995;182(2):389-400.
11. Inaba K, Inaba M, Romani N, Aya H, Deguchi M, Ikehara S, Muramatsu S, and Steinman RM. Generation of large numbers of dendritic cells from mouse bone marrow cultures supplemented with granulocyte/macrophage colony-stimulating factor. *J Exp Med*. 1992;176(6):1693-702.
12. Pisarev V, Yu B, Salup R, Sherman S, Altieri DC, and Gabrilovich DI. Full-length dominant-negative survivin for cancer immunotherapy. *Clin Cancer Res*. 2003;9(17):6523-33.
13. Schroers R, Shen, L., Slawin, K., Huang, XF, and Si-Yi Chen. Promiscuous MHC Class II-Restricted T-Helper Epitopes from Human Telomerase Reverse Transcriptase. *Clinical Can Res*. 2003;9(4743-55.
14. Schroers R, Huang XF, Hammer J, Zhang J, and Chen SY. Identification of HLA DR7-restricted Epitopes from Human Telomerase Reverse Transcriptase Recognized by CD4+ T-Helper Cells. *Cancer Res*. 2002;62(9):2600-5.
15. Schroers R, and Chen SY. Lentiviral transduction of human dendritic cells. *Methods Mol Biol*. 2004;246(451-9.
16. Schmidt SM, Schag K, Muller MR, Weck MM, Appel S, Kanz L, Grunebach F, and Brossart P. Survivin is a shared tumor-associated antigen expressed in a broad variety of malignancies and recognized by specific cytotoxic T cells. *Blood*. 2003;102(2):571-6.

Supplementary Fig. 1

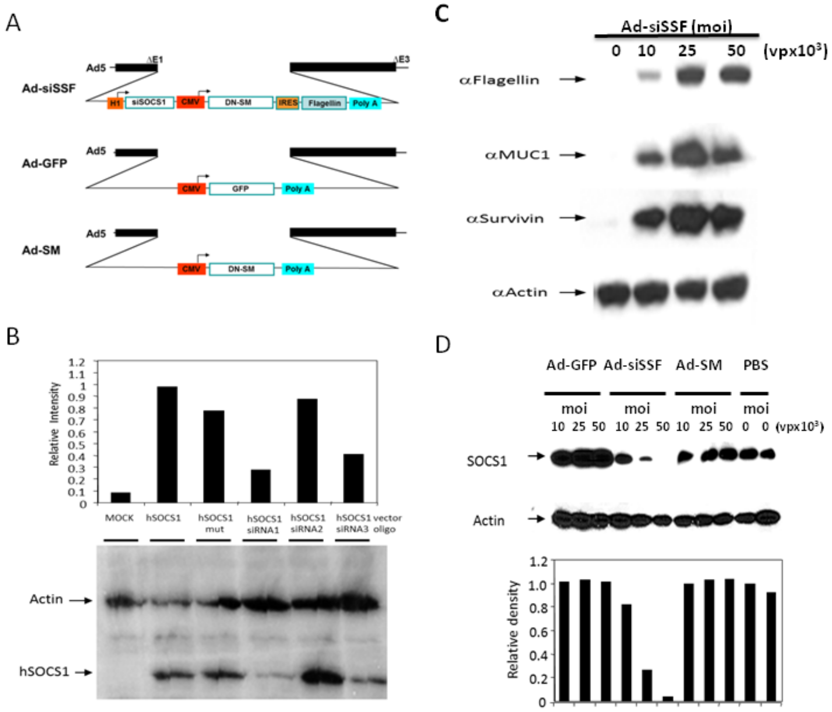

Supplementary Fig. 1. (A) Schematic representation of Ad-siSSFcoexpressing shSOCS1, dominant negative (DN) survivin and MUC1 fusion protein (SM), and secretory flagellin. (B) 293T cells were cotransfected with pCMV-hSOCS1 (0.4 µg) and synthetic siSOCS1 oligo duplexes (120 nmol/L) using GenePORTER. 48 hours later, the cell lysates were analyzed by western blotting. Relative levels of hSOCS1 and actin in each cell lysate were presented. (C). 293T cells were infected with the indicated moi of Ad-siSSF. Expression of Flagellin, Survivin, and Muc1 in the infected cells were detected by western blotting. (D). 293T cells were transfected

western blotting. Relative levels of hSOCS1 and actin in each cell lysate were presented. (C). 293T cells were infected with the indicated moi of Ad-siSSF. Expression of Flagellin, Survivin, and Muc1 in the infected cells were detected by western blotting. (D). 293T cells were transfected with pCMV-hSOCS1 (1 μg) followed by infection with the indicated moi of Ad-GFP, Ad-siSSF, Ad-SM, or PBS. 48 hours later, the cell lysates were analyzed by western blotting. Relative levels of hSOCS1 and actin in each cell lysate are presented, and increased moi of Ad-siSSF led to decreased levels of SOCS1. The experiments were repeated twice. VP: Viral Particle.

Supplementary Fig. 2

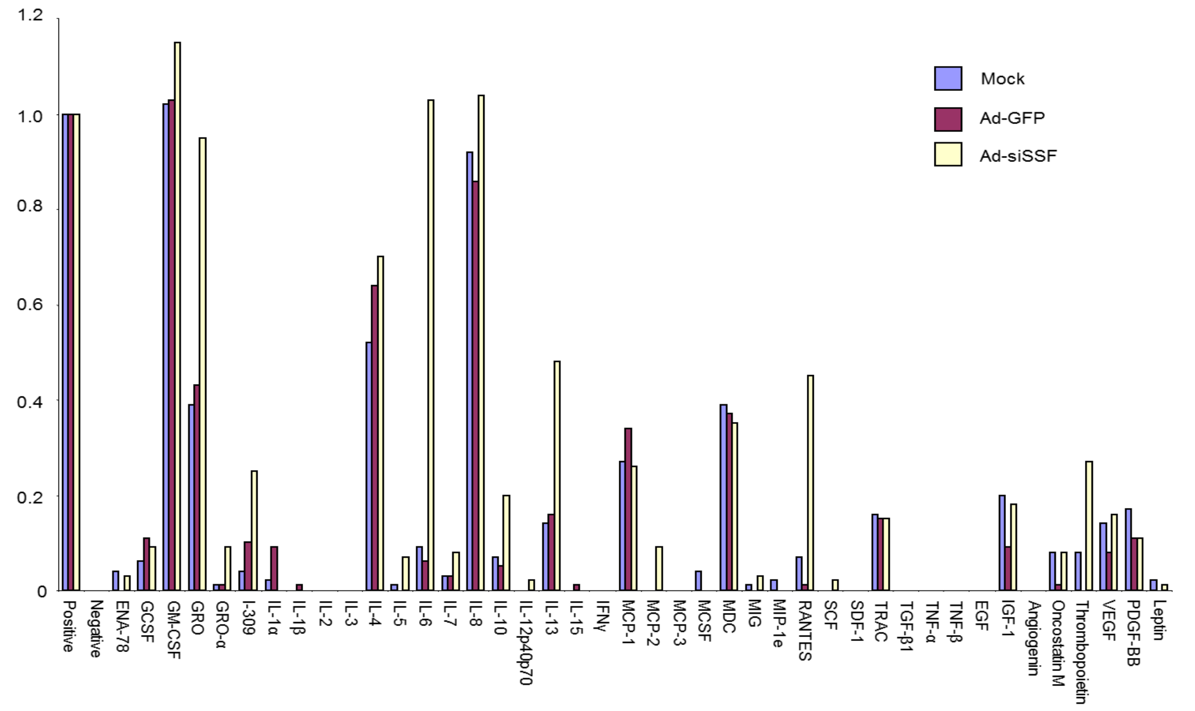

Supplementary Fig. 2.Ad-siSSF-transfected DCs exhibit increased secretion of various proinflammatory cytokines, detected by RayBio Human Cytokine Antibody Array 3 (Ray Biotech, Norcross, GA).

Supplementary Fig. 3

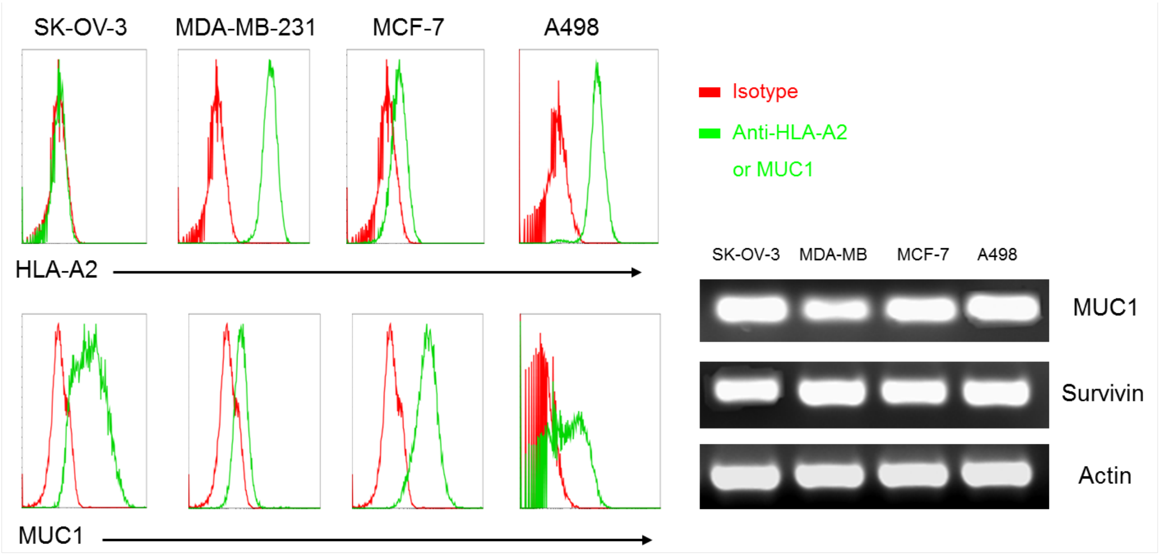

Supplementary Fig. 3. Flow cytometry analysis of HLA-A2 and MUC1 expression on human breast (MDA-MD-231 and MCF-7), renal (A498), and ovarian (SK-OV-3) tumor cells. MDA-MD-231 and MCF-7 were HLA-A2^{+} and MUC1^{+}, while SK-OV-3 were HLA-A2^{-} and MUC1^{+}.

breast (MDA-MD-231 and MCF-7), renal (A498), and ovarian (SK-OV-3) tumor cells. MDA-MD-231 and MCF-7 were HLA-$A2^+$ and $MUC1^+$, while SK-OV-3 were HLA-$A2^-$ and $MUC1^+$.

Supplementary Table 1. PCR primer sequences.

Gene	Primer sequence
Survivin (Thr34-Ala mutant)	F: 5'- GAGTCGAC**ATG**AAGGACCACCGCATCT-3'<br>R: 5'-ACCAAGCTTATCCATGGCAGCCAGCTG-3'
MUC1	F: 5'-ATTAACAAGCTTGGTGTCACCTCGGC-3'<br>R: 5'- TTAATTGCGGCCGCTTAGTGGGCTG-3'
Flagellin	F: 5’TAGTCGACCTCGAGATGCTCCTGGCTGTTTTGTACTGCCTGCTGTGG<br>AGTTTCCAGACCTCCGCTGGCCATTTCCCTAGAATGGCACAAGTCATTA-3’<br>R: 5’-GGCTCTAGAGCGGCCGCTTAACGCAGTAAAGAGAGG-3’
IRES	F: 5’-AATTGCGGCCGCTAAATTCCGCCCCTCT-3’<br>R: 5’-GGCCTCGAGTGTGGCCATATTATCATCG-3’
WT1	F: 5'-CAGGCTGCAATAAGAGATATTTTAAGCT-3'<br>R: 5'-GAAGTCACACTGGTATGGTTTCTCA-3' and<br>5'-CTTACAGATGCACAGCAGGAAGCACACTG-3'
ABL	F: 5'-TGGAGATAACACTCTAAGCATAACTAA AGGT-3'<br>R: 5'-GATGTAGTTGCTTGGGACCCA-3', and<br>5'-CCATTTTTGGTTTGGGCTTCACACCAT T-3'
